# *De Novo* Versus Secondary Metastatic *EGFR*-Mutated Non-Small-Cell Lung Cancer

**DOI:** 10.3389/fonc.2021.640048

**Published:** 2021-04-09

**Authors:** Farastuk Bozorgmehr, Daniel Kazdal, Inn Chung, Martina Kirchner, Nikolaus Magios, Mark Kriegsmann, Michael Allgäuer, Laura V. Klotz, Thomas Muley, Rami A. El Shafie, Jürgen R. Fischer, Martin Faehling, Albrecht Stenzinger, Michael Thomas, Petros Christopoulos

**Affiliations:** ^1^ Department of Thoracic Oncology, Thoraxklinik at Heidelberg University Hospital, Heidelberg, Germany; ^2^ Translational Lung Research Center Heidelberg (TLRC-H), Member of the German Center for Lung Research (DZL), Heidelberg, Germany; ^3^ Institute of Pathology, Heidelberg University Hospital, Heidelberg, Germany; ^4^ Department of Thoracic Surgery, Thoraxklinik at Heidelberg University Hospital, Heidelberg, Germany; ^5^ Translational Research Unit, Thoraxklinik at Heidelberg University Hospital, Heidelberg, Germany; ^6^ Department of Radiation Oncology, Heidelberg University Hospital, Heidelberg, Germany; ^7^ Department of Thoracic Oncology, Lungenklinik Löwenstein, Löwenstein, Germany; ^8^ Department of Cardiology, Angiology and Pneumology, Klinikum Esslingen, Esslingen, Germany

**Keywords:** EGFR^+^ NSCLC, *de novo*, secondary, prognosis, comutations, metastatic disease

## Abstract

**Background:**

Metastatic epidermal growth factor receptor-mutated (EGFR^+^) non-small-cell lung cancer (NSCLC) can present *de novo* or following previous nonmetastatic disease (secondary). Potential differences between these two patient subsets are unclear at present.

**Methods:**

We retrospectively analyzed characteristics of tyrosine kinase inhibitor (TKI)-treated patients with *de novo* vs. secondary metastatic EGFR^+^ NSCLC until December 2019 (n = 401).

**Results:**

*De novo* metastatic disease was 4× more frequent than secondary (n = 83/401), but no significant differences were noted regarding age (median 66 vs. 70 years), sex (65% vs. 65% females), smoking history (67% vs. 62% never/light-smokers), and histology (both >95% adenocarcinoma). Patients with secondary metastatic disease showed a better ECOG performance status (PS 0–1 67%–32% vs. 46%–52%, p = 0.003), fewer metastatic sites (mean 1.3 vs. 2.0, p < 0.001), and less frequent brain involvement (16% vs. 28%, p = 0.022) at the time of stage IV diagnosis. Progression-free survival (PFS) under TKI (median 17 for secondary vs. 12 months for *de novo*, p = 0.26) and overall survival (OS, 29 vs. 25 months, respectively, p = 0.47) were comparable. *EGFR* alterations (55% vs. 60% exon 19 deletions), *TP53* mutation rate at baseline (47% vs. 43%, n = 262), and T790M positivity at the time of TKI failure (51% vs. 56%, n = 193) were also similar. OS according to differing characteristics, e.g., presence or absence of brain metastases (19–20 or 30–31 months, respectively, p = 0.001), and ECOG PS 0 or 1 or 2 (32–34 or 20–23 or 5–7 months, respectively, p < 0.001), were almost identical for *de novo* and secondary metastatic disease.

**Conclusions:**

Despite the survival advantage reported in the pre-TKI era for relapsed NSCLC, molecular features and outcome of TKI-treated metastatic EGFR^+^ tumors are currently independent of preceding nonmetastatic disease. This simplifies design of outcome studies and can assist prognostic considerations in everyday management of patients with secondary metastatic EGFR^+^ tumors.

## Introduction

Epidermal growth factor receptor-mutated non-small-cell lung cancer (EGFR^+^ NSCLC) comprises about 10%–15% of lung adenocarcinomas (1). According to the current guidelines, the preferred therapy for metastatic disease are tyrosine kinase inhibitors (TKI), which have consistently shown superior efficacy and better tolerability than chemotherapy (2). In contrast, treatment of earlier-stage tumors relies on other modalities, such as surgery, radiotherapy, chemotherapy, and/or immunotherapy, the use of which does not depend on the results of molecular tumor work-up (2). Consequently, testing for *EGFR* mutations is currently standard practice only for stage IV or otherwise incurable NSCLC (3), which results in two distinct subpopulations of these patients: “*de novo*” cases that directly present with stage IV disease, and “secondary” metastatic EGFR^+^ NSCLC cases that are initially diagnosed with stage I-III disease and develop stage IV NSCLC through subsequent relapse or progression, upon which molecular analysis is performed and *EGFR* mutations are detected. From a practical standpoint, both patient subsets are currently being treated according to the same principles (2), but potential clinical and biologic differences remain unclear. In addition, several factors, such as the metastatic pattern, histological subtype, *EGFR* variant, presence of *TP53* mutations, ECOG performance (PS) and smoking status have been shown to correlate with survival of stage IV EGFR^+^ lung cancer patients (4–9), but it is unknown whether the distribution and the prognostic importance of these parameters are similar between *de novo* and secondary metastatic cases. The current study was performed to address these topics and is presented in accordance with the STROBE reporting checklist.

## Material and Methods

### Patient Selection and Clinical Data

This retrospective study was approved by the ethics committee of Heidelberg University (S-145/2017) and included all 401 stage IV NSCLC patients with sensitizing mutations of *EGFR* exons 18–21 treated with TKI at the Thoraxklinik Heidelberg and its regional network hospitals (Löwenstein, Esslingen) between 2010 and 2019. Patients with *EGFR* exon 20 insertions (n = 39) were excluded, since they respond poorly to currently approved targeted compounds and are mainly managed with other treatments (10). Histological diagnosis of early, as well as histological diagnosis and molecular profiling of metastatic NSCLC were performed at the Institute of Pathology Heidelberg using next-generation sequencing (NGS) after September 2014 (n = 262), and Sanger sequencing previously, as described (1, 11, 12). For NGS, a semiconductor-based platform (ThermoFisher Scientific, Waltham, MA, USA) was used with a custom panel covering 38–42 genes considered relevant for lung cancer biology, which included all *EGFR* exons, *TP53* exons 4–10, *CDKN2A* exons 1–2, *PIK3CA* exons 2, 5, 8 10, 14, 21, and *CTTNB1* exon 3 (**Table 1**), as published (1). Clinical data were collected through a systematic review of patient records with a cut-off on December 31 2019. Overall survival (OS) was calculated from the start of systemic treatment for stage IV disease. Progression-free survival (PFS) was calculated from the first dose of TKI or chemotherapy administered for metastatic disease until radiologic disease progression or death, whichever occurred first. Cross-sectional imaging studies, i.e., chest and abdomen CT/brain MRI were routinely performed at baseline and every 6 to 12 weeks thereafter, or earlier in case of clinically suspected progression. The progression date was verified through review of radiologic images by the investigators without formal RECIST reassessment, since progression based on real-world data has been shown to correlate reasonably well with RECIST-defined progression in recent studies (13, 14). Patients initially diagnosed with early tumors that preceded development of secondary metastatic EGFR^+^ NSCLC, had undergone regular follow-up including imaging studies every 3 months during the first 2 years, every 6 months in years 3–5, and yearly thereafter as per our institutional standard, which was taken into account in the interpretation of results.

**Table 1:** Patient characteristics.

All stage IV EGFR^+^ NSCLC patients (n = 401, 100%)		*de novo* St. IV (n = 318)	secondary St. IV (n = 83)	p-value ^1^
**Clinical characteristics at diagnosis of stage IV**				
Age, median (IQR)		66 (18)	70 (16)	p = 0.248
Gender, % female (n)		65 (208)	66 (55)	p = 0.883
Smoking status, % (n) ^2^	never/light smokers	67 (212)	62 (50)	p = 0.344
ECOG PS, % (n)	0	46 (137)	67 (53)	p = 0.003
	1	52 (155)	32 (25)	
	2	3 (8)	1 (1)	
**Pathological and molecular characteristics**				
Histology, % (n)	adenocarcinoma	97 (307) 60 (192) 28 (88) 12 (38)	96 (80) 55 (46) 30 (25) 14 (12)	p =0.646 p =0.689
EGFR alteration (%)	exon 19del/ins L858R rare mutations			
NGS workup at baseline, % (n)		66 (211)	61 (51)	p = 0.403
any co-mutation at diagnosis of stage IV, % (n)		59 (124/211)	65 (33/51)	p = 0.437
*- TP53* co-mutation at diagnosis of stage IV, % (n)		43 (90/211)	47 (24/51)	p = 0.569
*- CTTNB1* co-mutation at diagnosis of stage IV, % (n)		4 (8/211)	4 (2/51)	p = 0.965
*- CDKN2A* co-mutation at diagnosis of stage IV, % (n)		3 (7/211)	8 (4/51)	p = 0.148
*- PIK3CA* co-mutation at diagnosis of stage IV, % (n)		3 (6/211)	6 (3/51)	p = 0.285
- co-mutation of any other gene in the NGS panel ^3^		< 2%	< 2%	
*EGFR* T790M positivity at time of TKI failure, % (n) ^4^		56 (87/156)	51 (19/37)	p = 0.658
**Treatment for stage IV disease**				
first TKI treatment, % (n)	first line	73 (231)	86 (71)	p = 0.061
	second line	25 (80)	13 (11)
	third line and beyond	2 (5)	1 (1)
first TKI compound, % (n)	afatinib	19 (59)	19 (16)	p = 0.316
	erlotinib	38 (122)	48 (40)	
	gefitinib	30 (97)	22 (18)	
	osimertinib	13 (40)	11 (9)	
CHT administration, % (n)	platinum doublet	31 (98)	55 (46)	p=0.113
	monotherapy	8 (24)	6 (5)	
palliative radiotherapy, % (n)		51 (161)	43 (36)	p=0.239
**Patient survival**				
OS from treatment start for stage IV, median (IQR)		25 (20–29)	29 (23–35)	p = 0.466
PFS for the first TKI line, median in months (IQR)		12 (7–21)	17 (8–27)	p = 0.259
CHT PFS, median in months (IQR)		6 (3–8)	4 (2–9)	p = 0.744
Follow-up duration, median in months (IQR)		38 (17–63)	37 (20–65)	p = 0.761

IQR, interquartile range; NGS, next-generation sequencing; PS, performance status; TKI, tyrosine kinase inhibitor; CHT, chemotherapy; PFS, progression-free survival; OS, overall survival.

^1^Statistical comparisons with a chi-square test for categorical, t-test for numerical, and logrank test for time-to-event variables.

^2^Smoking status available for 315/318 and 81/83 cases; “never/light-smokers” refers to <10 pack-years; ECOG PS available for 300/318 and 79/83 cases.

^3^In alphabetical order: ACVR2A, AKT, ALK, APC, ARID1A, BRAF, CBL, CCND1, CCNE1, CD274, CDK6, DDR2, ERBB2, ERBB4, EYS, AMER1, FBXW7, FGFR1/2/3, HRAS, JAK2, KEAP1, KIT, KRAS, MEK1, MCL-1, MDM2, MET, MSH3, MYC, NFE2L2, NKX2-1, NOTCH1, NRAS, PDGFRA, PIK3R1, POLE, PTEN, RB1, RBM10, RET, EIT1, ROS1, SMAD4, SMARCA4, SXO2, STK11, TCF7L2, TERT, U2AF1, as published in (1).

^4^T790M testing was performed for 193 cases overall (113 with tissue rebiopsies, 60 with liquid biopsies, and 20 with both).

### Statistical Analysis

The effect of categorical and continuous parameters on survival was analyzed according to Kaplan-Meier with logrank tests and Cox regression analysis. Duration of follow-up was calculated using the reverse Kaplan-Meier method. Numerical data were compared among two groups with the Student’s t-test, while for categorical data the chi-square test was used. All statistical calculations were performed with SPSS version 24 (IBM, Armonk, NY, USA), while plots were generated with GraphPad Prism version 7 (La Jolla, CA, USA).

## Results

### Patient Baseline Characteristics

Among the entire population of 401 study patients, 318 were diagnosed with primary stage IV disease (79%, “*de novo*” stage IV EGFR^+^ NSCLC, **Table 1**), while 83 had initially presented with nonmetastatic disease, were treated as shown in **Table 2**, and developed stage IV EGFR^+^ NSCLC after subsequent tumor relapse or progression (21%, “secondary” stage IV). Demographics of both patient groups were very similar. The median age at diagnosis was 66 years [interquartile range (IQR) 18] for *de novo*, and 70 years (IQR 16) for secondary metastatic disease (**Table 1**). About two-thirds of the patients in each group were females (65% and 66%) and never/light-smokers (i.e., had tobacco exposure <10 pack-years, 67% and 62%, **Table 1**). The vast majority were diagnosed with adenocarcinoma (>95% in both groups, **Table 1**). *EGFR* variants showed similar distributions in the two groups (60% vs. 55% exon 19 deletions [del19], 28% vs. 30% L858R, and 12% vs. 14% rare mutations, **Table 1**). Within the subset of patients with available NGS profiling at baseline (262/401, i.e., patients tested after implementation of NGS in 2014, see *Material and Methods*), the two patient groups showed a similar frequency of co-mutations in *TP53* (43% vs. 47%) and other genes included in our panel (39 vs. 40%, **Table 1**).

**Table 2:** Treatment of nonmetastatic EGFR^+^ tumors preceding secondary stage IV disease.

All patients with secondary stage IV EGFR^+^ NSCLC (n = 83)					
Initial stage	**% of total (n)**	Surgery **% of stage (n)**	SRT primary **% of stage (n)**	CRT primary **% of stage (n)**	Time to stage IV **months, mean (SE)**
**stage I**	22 (18)	94 (17) ^1^	6 (1)	–	36 (9)
**stage II**	14 (12)	92 (11) ^2^	8 (1)	–	35 (6)
**stage III**	64 (53) ^3^	68 (36) ^4,5^	2 (1)	19 (10) ^6^	19 (3)

SRT, stereotactic radiotherapy; CRT, chemoradiotherapy.

^1^3/15 patients also subsequently received thoracic irradiation due to a localized relapse of the tumor before the systemic relapse leading to secondary stage IV disease.

^2^10/11 patients received adjuvant chemotherapy, while in 1/11 cases (pT3pN0) this was omitted due to multiple comorbidities and absence of lymph node metastases.

^3^6/53 patients started with inductive systemic therapy (4/6 with chemotherapy, and 2/6 with TKI due to comorbidities and patient preference), but did not receive subsequent local treatment because of disease progression (2/6), complications (1/6) or patient refusal (3/6).

^4^23/36 patients received adjuvant chemotherapy; reasons for not giving it were postoperative complications or comorbidities in 7/36, progressive disease after postoperative radiotherapy and before adjuvant chemotherapy could be started in 4/36, and patient refusal in 2/36 (NB. postoperative radiotherapy was given prior to chemotherapy in these cases due to R1 resection).

^5^14/36 patients received postoperative radiotherapy due to R1 resection and/or nodal involvement.

^6^3/10 simultaneous chemoradiotherapy, 7/10 sequential chemoradiotherapy, in all cases with a platinum-based doublet.

**Figure 1:**
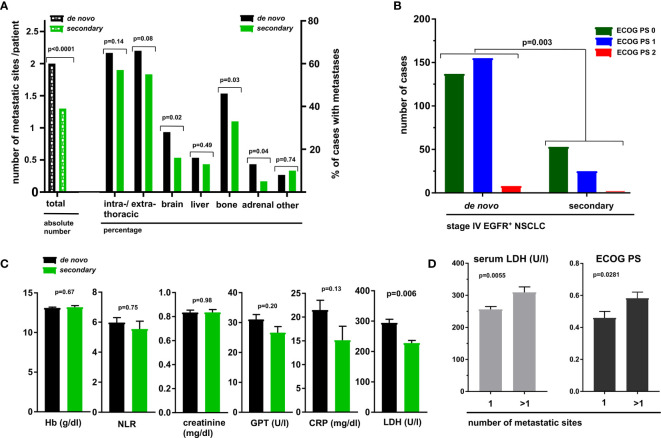
Metastatic spread, ECOG performance status (PS), and basic laboratory values at diagnosis of *de novo* vs. secondary metastatic EGFR^+^ NSCLC. **(A)** The average number of metastatic sites (lung, pleura, brain, liver, bone, adrenals, other) and the percentage of cases with metastases for each localization in *de novo* vs. metastatic stage IV EGFR^+^ NSCLC. Statistical comparisons were performed with the Student’s t-test for numerical (left part), and with the chi-square test for categorical data (right part). Details are shown in **Supplementary Table 1**. **(B)** The distribution of ECOG PS among patients diagnosed with *de novo* vs. secondary stage IV EGFR^+^ NSCLC, compared with a chi-square test. Details are shown in **Table 1**. **(C)** Results of basic laboratory tests at diagnosis of *de novo* vs. secondary stage IV EGFR^+^ NSCLC, compared with the Student’s t-test. Boxes and error bars show mean values and standard errors. Details are given in **Supplementary Table 2**. **(D)** Relationship between serum LDH and the number of metastatic sites at diagnosis of stage IV EGFR^+^ NSCLC (p = 0.006 with the Student’s t-test). Boxes and error bars show mean values and standard errors. Details are given in **Supplementary Table 2**. Relationship between the ECOG PS and the number of metastatic sites at diagnosis of stage IV EGFR^+^ NSCLC (p = 0.028 with the Student’s t-test). Boxes and error bars show mean values and standard errors, respectively. Details are given in **Table 1**.

One first difference was the higher total number of metastatic sites at the time of stage IV diagnosis in *de novo* compared to secondary cases (mean 2.0 vs. 1.3, p < 0.001, **Figure 1A** and **Supplementary Table 1**). Both intra- and extrathoracic metastases tended to be increased in *de novo* stage IV cases to a similar extent, with significant differences in the frequency of brain (28% vs. 16%, p = 0.022), bone (46% vs. 33%, p = 0.032) and adrenal lesions (13% vs. 5%, p = 0.038, **Figure 1A** and **Supplementary Table 1**). In addition, at the time of stage IV diagnosis, patients with *de novo* metastatic disease had a worse ECOG performance status (PS 0-1-2 rates 46%-52%-3% vs. 67%–32%, respectively, p = 0.003), i.e., presentation with initially nonmetastatic disease was linked to a better ECOG PS at the time of subsequent stage IV relapse compared to patients with *de novo* stage IV (**Figure 1B** and **Table 1**). Basic laboratory parameters, such as the blood neutrophil-to-lymphocyte ratio (NLR), blood hemoglobin, blood platelet counts, serum C-reactive protein (CRP), serum creatinine, and serum GPT were similar among the two groups, with the notable exception of serum lactate dehydrogenase (LDH), which was increased in patients with *de novo* compared to secondary metastatic disease (mean 295 vs. 228 U/l, p = 0.006, **Figure 1C** and **Supplementary Table 2**). Both the ECOG PS and the level of serum LDH were significantly associated with the number of metastatic sites at diagnosis of stage IV disease (**Figure 1D**).

### Patient Treatment and Survival

Treatment characteristics for the two groups of patients with metastatic EGFR^+^ NSCLC were very similar (**Table 1**). All cases received EGFR TKI, mostly first/second-generation compounds (87% vs. 89% for *de novo* vs. secondary), which were administered as the first palliative treatment line in over two-thirds of patients (73% and 86%, respectively). For patients with molecular reanalysis in a tissue or liquid rebiopsy at the time of TKI failure, rates of T790M positivity were also similar (56% vs. 51%, **Table 1**). Moreover, the frequencies of palliative radiotherapy (51% vs. 43%), platinum doublets (31% vs. 55%), or mono-chemotherapy (8% vs. 6%) did not show significant differences (**Table 1**).

Patient survival was also comparable for *de novo* vs. secondary metastatic cases: OS from the start of treatment for stage IV disease (25 vs. 29 months in median, p = 0.47), PFS for the first TKI treatment line (12 vs. 17 months in median, p = 0.26), and PFS for the first chemotherapy treatment line (6 vs. 4 months in median, p = 0.74) did not differ substantially between the two groups (**Table 1**). For the subset of patients with secondary tumors after surgery and adjuvant chemotherapy (n = 33), the molecular profile and survival were also comparable to these of patients with *de novo* stage IV tumors (**Supplementary Table 3**).

### Influence of *De Novo* vs. Secondary Setting on the Impact of Prognostic Factors in Metastatic EGFR^+^ Non-Small-Cell Lung Cancer

**Figure 2:**
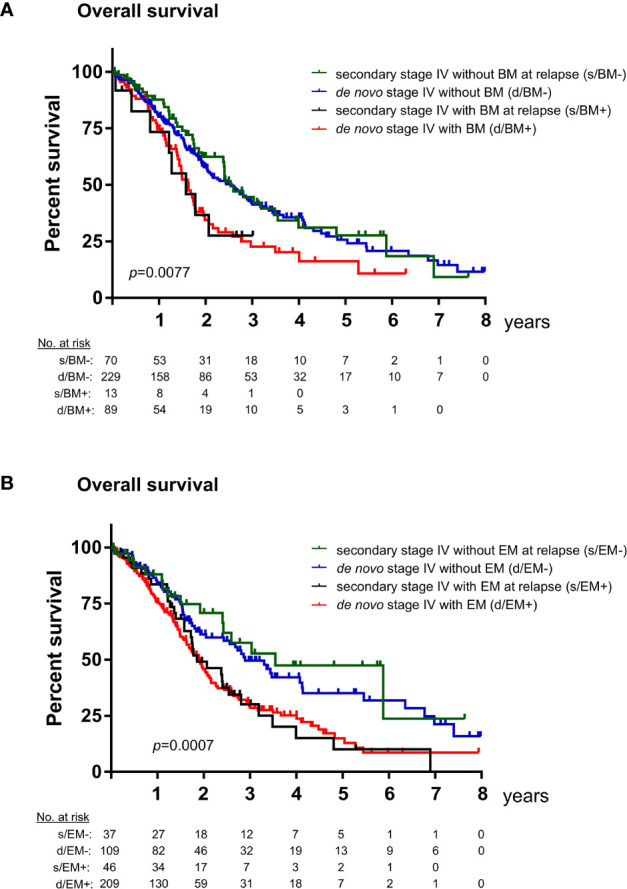
Overall survival of TKI-treated EGFR^+^ NSCLC patients with *de novo* vs. secondary metastatic disease by the presence of brain and extrathoracic metastases. **(A)** Median overall survival (OS) from the diagnosis of metastatic disease for TKI-treated EGFR^+^ NSCLC was 30 months (standard error [SE] 2.5) for patients with secondary stage IV disease without brain metastases (BM, n = 70), 31 months (SE 3.3) for patients with *de novo* stage IV disease without BM (n = 229), 19 months (SE 5.0) for patients with secondary stage IV disease and BM (n = 13), and 20 months (SE 1.5) for patients with *de novo* stage IV disease and BM (n = 89, logrank p = 0.008). In a bivariable Cox regression, only presence of brain metastases at diagnosis of stage IV disease (hazard ratio [HR] = 1.65, p = 0.001), but not presence of *de novo* vs. secondary metastatic disease (HR 1.06, p = 0.75) was a significant predictor for OS in our entire cohort (n = 401). **(B)** Median OS from the diagnosis of metastatic disease for TKI-treated EGFR^+^ NSCLC was 43 months (SE 12) for patients with secondary stage IV disease without extrathoracic metastases (EM, n = 37), 35 months (SE 4.4) for patients with *de novo* stage IV disease without EM (n = 109), 22 months (SE 4) for patients with secondary stage IV disease and EM (n = 46), and 23 months (SE 1.5) for patients with *de novo* stage IV disease with EM (n = 89, logrank p < 0.001). In a bivariable Cox regression, only presence of extrathoracic metastases at diagnosis of stage IV disease (HR = 1.79, p < 0.001), but not presence of *de novo* vs. secondary metastatic disease (HR = 1.09, p = 0.62) was a significant predictor for OS in our entire cohort (n = 401).

**Figure 3:**
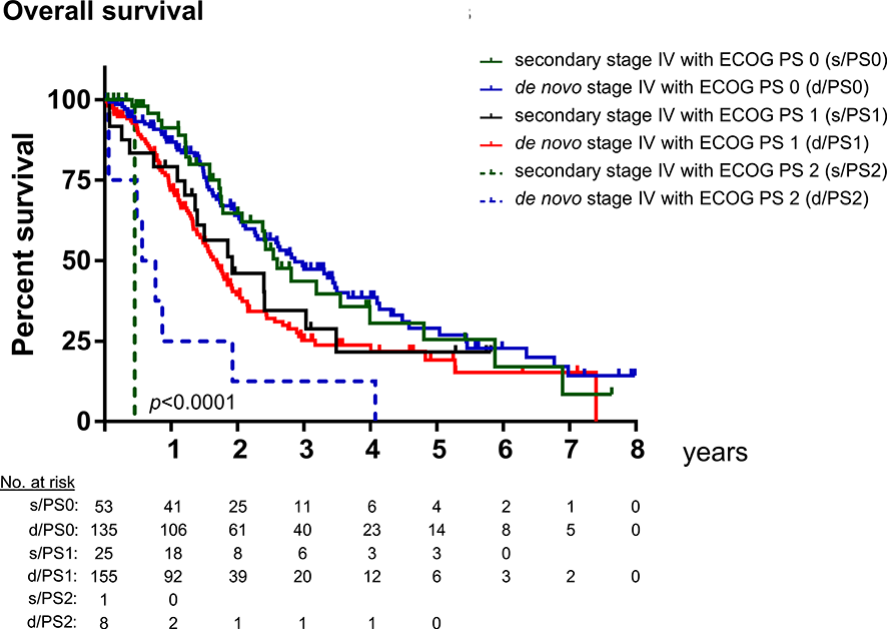
Overall survival of TKI-treated EGFR^+^ NSCLC patients with *de novo* vs. secondary metastatic disease by the ECOG performance status. Median overall survival (OS) from the diagnosis of metastatic disease for TKI-treated EGFR^+^ NSCLC was 32 months (standard error [SE] 3.0) for patients with secondary stage IV disease and ECOG performance status 0 (PS 0, n = 53), and 34 months (SE 4.3) for patients with *de novo* stage IV disease and ECOG PS 0 (n = 135); 23 months (SE 7.2) for patients with secondary stage IV disease and ECOG PS 1 (n = 25), and 20 months (SE 1.5) for patients with *de novo* stage IV disease with EM (n = 154); 5 months for the single patient with secondary stage IV disease and ECOG PS 2 (n = 1), and 6.7 months (SE 2.4) for patients with *de novo* stage IV disease and ECOG PS 2 (n = 8, logrank p < 0.0001). In a bivariable Cox regression, only the ECOG PS at diagnosis of stage IV disease (HR = 1.66, p < 0.0001 for ECOG PS 1; HR = 4.65, p < 0.0001 for ECOG PS 2), but not presence of *de novo* vs. secondary metastatic disease (HR = 1.02, p = 0.91) was a significant predictor for OS in our entire cohort (n = 401).

The previous comparisons revealed two main differences in the basic clinical characteristics of patients with *de novo* vs. secondary metastatic EGFR^+^ NSCLC: frequency of brain and other extrathoracic metastases, as well as ECOG PS at diagnosis of stage IV disease (**Table 1** and **Supplementary Table 1**). Since these factors have been reported to correlate with survival of stage IV EGFR^+^ NSCLC patients, we examined whether this association might be affected by the setting of metastatic disease, i.e., *de novo* vs. secondary. The presence of brain metastases was associated with almost identical OS regardless of whether the metastatic disease was *de novo* or secondary (31 or 30 months in median, respectively, for cases without brain metastases, and 20 or 19 months for cases with brain metastases, **Figure 2A**). A bivariable Cox regression was additionally performed in order to obtain a quantitative measure for the effect of brain involvement on OS (hazard ratio (HR) = 1.65, p = 0.001) relative to that of the metastatic disease setting (HR = 1.06, p = 0.75, **Table 3**). Likewise, OS was strongly associated with the presence of extrathoracic metastases (HR = 1.79 with p < 0.001, vs. HR = 1.09 with p = 0.62, **Figure 2B** and **Table 3**) and ECOG PS at the time of stage IV diagnosis (HR = 1.66 with p < 0.001 for PS 1, and HR = 4.65 with p < 0.001 for PS 2, vs. HR = 1.02 with p = 0.91, **Figure 3** and **Table 3**) regardless of whether the metastatic disease was *de novo* or secondary.

**Table 3:** Relative effect of the *de novo* vs. secondary setting, compared to the effect of metastatic pattern and ECOG performance status on overall survival of EGFR^+^ lung cancer patients.

prognostic impact relative to that of the metastatic setting:	OS from start of treatment for stage IV disease	
- for presence or absence of brain metastases:	Cox HR	95% CI
***de novo* vs. secondary metastatic setting**	1.06 p = 0.745	0.76–1.47
**presence of brain metastases**	1.65 p = 0.001	1.23–2.23
- for presence or absence of extrathoracic metastases:		
***de novo* vs. secondary metastatic setting**	1.09 p = 0.624	0.78–1.50
**presence of extrathoracic metastases**	1.79 p < 0.001	1.34–2.38
- for ECOG PS at diagnosis of stage IV:		
***de novo* vs. secondary metastatic setting**	1.02 p = 0.907	0.73–1.42
**ECOG PS 0 (reference)**		
**ECOG PS 1**	1.66 p < 0.0001	1.26–2.18
**ECOG PS 2**	4.65 p < 0.0001	2.33–9.27

The association of brain metastases, extrathoracic metastases, and baseline ECOG performance status (PS) with overall survival (OS) of stage IV EGFR^+^ lung cancer patients was analyzed including the metastatic disease setting (de novo vs. secondary) as an additional independent factor. ECOG PS was included in the bivariable Cox regression as a categorical variable.

HR, hazard ratio; 95% CI, 95% confidence interval.

## Discussion

Metastatic EGFR^+^ NSCLC is a model disease for the efficacy of targeted therapies in thoracic oncology. Upfront administration of increasingly potent TKI, meanwhile spanning three generations, has considerably improved prognosis with the median overall survival currently approaching 3 years (5, 15–17). However, clinical courses of individual patients can vary widely in association with several clinical and pathological parameters (4–9). The present study analyzed the potential impact that a prior diagnosis with stage I-III disease might have on the clinical course of stage IV EGFR^+^ NSCLC patients.

A first important finding is that the setting of stage IV disease, *de novo* vs. secondary, itself is not associated with survival of EGFR^+^ NSCLC patients from the time of stage IV diagnosis: PFS under TKI or chemotherapy, and OS were similar among the two patient groups (**Table 1**). Furthermore, most other parameters known to be associated with OS of stage IV EGFR^+^ NSCLC, such as the histological subtype, type of *EGFR* mutation, *TP53* status, presence of co-mutations, especially in *TP53*, frequency of T790M development (5–7, 9), were equally distributed among *de novo* and secondary cases (**Table 1**). Along the same lines, a previous study observed a similar distribution of *EGFR* mutations among early-stage and metastatic EGFR^+^ lung cancers (18), which is also consistent with the notion that *EGFR* alterations represent “early events” during lung carcinogenesis (19). Moreover, other investigators have noted that the tumor mutational burden of NSCLC does not change after relapse of resected early-stage disease (20), or after chemotherapy and radiotherapy (21). The only significant differences seen in our study were: i) the extent of metastatic spread, with more frequent involvement of brain and other extrathoracic sites, as well as higher serum LDH values among *de novo* vs. secondary cases (**Figure 1**, **Supplementary Tables 1** and **2**); and ii) the ECOG performance status, which was also worse among *de novo* cases (**Table 1**). Interestingly, even though both the metastatic pattern and ECOG PS have been described as predictors of survival in metastatic EGFR^+^ NSCLC (5, 9, 22), our results show clearly that their associations with OS are not affected by the *de novo* vs. secondary metastatic setting (**Figures 2** and **3**). Therefore, a first conclusion is that *de novo* and secondary metastatic cases can be considered together when analyzing the effect of all currently known prognostic factors in TKI-treated EGFR^+^ NSCLC.

A second important conclusion is that the “left-shift” toward more limited metastatic spread within stage IV, evident as fewer metastatic sites, lower serum LDH and a better ECOG PS for “secondary” compared to *de novo* cases (**Figure 1D**), does not have a significant impact on the outcome of TKI-treated EGFR^+^ NSCLC (**Table 1**). In keeping with this, an earlier study had found no relationship between initial tumor volume and OS of TKI-treated EGFR^+^ NSCLC patients (23). This contrasts the results of several previous investigations including all-comer and/or non-TKI-treated NSCLC cohorts [(24) and (25) both published in 2019, but analyzing patients diagnosed until 2012 and 2013, respectively; (26) analyzing EGFR^+^ patients diagnosed until 2011], which have observed a slight, but significant OS advantage in the “secondary” setting. In our cohort this advantage is absent, presumably neutralized by the much higher efficacy of TKI, which all our stage IV patients per inclusion criteria received, compared to the chemotherapy-only treatment in the aforementioned previous studies. Thus, our findings aid correct interpretation of the published literature by extending and updating older results in the context of contemporary treatment for EGFR^+^ lung cancer.

The similar benefit (PFS) from TKI and chemotherapy, the comparable OS, and the absence of other clinical, laboratory or molecular disparities between secondary and *de novo* metastatic EGFR^+^ NSCLC (**Table 1**) argue against true biologic differences among them. Instead, the observed lower tumor burden of secondary cases (**Figure 1**) is probably simply due to an earlier diagnosis, because patients treated for early-stage disease undergo regular imaging follow-up, which can detect subsequent relapse or progression even if asymptomatic. Along the same lines, a more limited metastatic spread with less extrapulmonary metastatic sites, as well as a better ECOG PS for secondary (relapsed) vs. *de novo* metastatic tumors have also been noted by other investigators, who analyzed unselected NSCLC patients regardless of *EGFR* mutation status (24, 25). One limitation of our study is the lack of detailed data about patient symptoms at the time of diagnosis. However, the better ECOG PS of secondary metastatic cases at baseline (**Figure 1** and **Table 1**) suggests that secondary tumors were indeed detected despite being less symptomatic than their *de novo* counterparts. Of note, the ratio of secondary to *de novo* metastatic EGFR^+^ NSCLC, approximately 1:4 in our study (**Table 1**), itself is not a purely biological characteristic either, but also influenced by the efficacy of lung cancer detection strategies. For example, the upcoming implementation of volume CT-based lung cancer screening according to the NELSON findings, is expected to increase this ratio, since many tumors previously detected as *de novo* metastatic NSCLC will then be diagnosed already in earlier stages, and also proportionally contribute more to the pool of “secondary” stage IV after relapse (27). Nevertheless, according to the results of the current study, across the entire population of patients ultimately developing stage IV EGFR^+^ NSCLC, the prognosis is not expected to change significantly.

The apparent lack of a specific biologic basis for the higher metastatic burden of *de novo* vs. secondary metastatic EGFR^+^ NSCLC contrasts a similar constellation in the closely related ALK^+^ NSCLC: here, an increased number of metastatic sites at initial diagnosis is also associated with a worse baseline ECOG PS (28), but linked to the special biologic properties of *EML4-ALK* variant 3 (V3) that enhance cancer dissemination (28–31). Consequently, several well-controlled retrospective analyses (32–35) and the prospective phase 3 clinical trial ALTA-1L (36) have demonstrated a more aggressive clinical course and shorter survival for *EML4-ALK* V3^+^ patients, while metastatic EGFR^+^ NSCLC appears to be prognostically homogenous regardless of the metastatic disparities between the *de novo* and secondary setting. At the same time, this similarity of secondary cases to their *de novo* counterparts, suggests that paired analysis of secondary stage IV tumors comparative to their earlier stage ancestors represents a promising approach to decipher the molecular pathogenesis of metastatic EGFR^+^ lung cancer in general. The particular molecular features of early EGFR^+^ lung cancers and their potential impact on TKI efficacy now acquire direct clinical relevance due to the recent promising results of the ADAURA study, which might lead to routine adjuvant administration of osimertinib in the near future (37).

From a practical standpoint, an important consequence of the results presented in this study is that patients with *de novo* and secondary TKI-treated metastatic EGFR^+^ NSCLC can be safely considered together in various analyses, in contrast to concerns regarding non-TKI-treated NSCLC based on earlier investigations (24–26). For example, one potential application is the development of risk stratification schemes for EGFR^+^ NSCLC, for which the data of both patient subsets could be merged together in order to derive an algorithm that will then be equally applicable to both. A second relevant issue are decisions about patient stratification in clinical trials: for example, when faced with the dilemma of whether to stratify newly diagnosed stage IV EGFR^+^ NSCLC patients according to the presence or absence of prior early-stage disease, or according to their current brain or ECOG PS status, our results show that stratification according to the baseline characteristics of the current stage IV disease is more important, and actually sufficient (**Figures 2** and **3**, **Table 3**). Main limitation of our work is its retrospective character, which cannot guarantee the absence of potential confounders. However, the relatively large size, homogeneity and typical characteristics of our cohort, the simple study design, the long follow-up, and the clear results add to the quality of the evidence presented by this study and suggest generalizability.

In summary, the present study shows that clinical and molecular features of metastatic EGFR^+^ NSCLC are largely independent of preceding nonmetastatic disease. This insight aids interpretation of older findings by updating them in the contemporary therapeutic context, simplifies the design of prospective or retrospective outcome studies, and can support prognostic considerations in everyday management of patients with secondary metastatic disease.

## Data Availability Statement

The datasets generated and analyzed in this study are available from the corresponding author upon reasonable request.

## Ethics Statement

This study was reviewed and approved by the ethics committee of the Heidelberg University (approval S-145/2017). Since this was a non-interventional, retrospective study, informed consent was obtained whenever possible, but its need for every participant was waived by the ethics committee.

## Author Contributions

FB and PC conceptualized and designed the study. IC, AS, MT, and PC provided administrative support. FB, NM, LK, JF, MF, MT, and PC provided the study materials or patients. FB, DK, IC, MKi, NM, MKr, MA, TM, RE, JF, MF, MT, and PC collected and assembled the data. FB, DK, AS, MT, and PC analyzed and interpreted the data. All authors contributed to the article and approved the submitted version.

## Funding

This study was supported by the German Center for Lung Research (DZL). The funding source did not have any influence on the design, conduction, and report of the results for this study.

## Conflict of Interest

FB reports honoraria from Novartis, MSD, ChugaiPharma, Roche, AstraZeneca and research grants from AstraZeneca, BMS, and Roche. DK reports personal fees from AstraZeneca, personal fees from Bristol-Myers Squibb GmbH, and personal fees from Pfizer Pharma GmbH. TM reports research funding from Roche and patents with Roche. JF reports advisory board honoraria from Boehringer, Roche, Celgene, and AstraZeneca. AS reports advisory board honoraria and/or speaker fees: Astra Zeneca, Bayer, Eli Lilly, Roche, BMS, Illumina, MSD, Novartis, Pfizer, Seattle Genetics, Takeda, Thermo Fisher, and research grants from BMS, Bayer, and Chugai. MT reports advisory board honoraria from Novartis, Lilly, BMS, MSD, Roche, Celgene, Takeda, AbbVie, Boehringer, speaker’s honoraria from Lilly, MSD, Takeda, research funding from AstraZeneca, BMS, Celgene, Novartis, Roche, and travel grants from BMS, MSD, Novartis, Boehringer. PC reports lecture/advisory board fees from AstraZeneca, Boehringer, Chugai, Novartis, Pfizer, Roche, and Takeda, as well as research funding from AstraZeneca, Novartis, Roche, and Takeda.

The remaining authors declare that the research was conducted in the absence of any commercial or financial relationships that could be construed as a potential conflict of interest. 
