# Supplementary material for: De Novo Versus Secondary Metastatic EGFR-Mutated Non-Small-Cell Lung Cancer
Source: Front Oncol. 2021 Apr 9;11:640048. doi: 10.3389/fonc.2021.640048 (PMC8063726; doi:10.3389/fonc.2021.640048)
Supplement: Supplementary file 1 [file DataSheet_1.pdf]

frontiers

Supplementary Material

Supplementary Tables

Supplementary Table 1: Number of metastatic sites at diagnosis in the study population

All stage IV $EGFR^+$ NSCLC patients (n=401, 100%)	*de novo* St. IV (n=318)	secondary St. IV (n=83)	p-value [1]
Number of metastatic sites, mean (SE)	2.0 (1.0)	1.3 (0.7)	p<0.001
- intrathoracic metastases, % (n) [2]	65 (208)	57 (47)	p=0.139
- extrathoracic metastases, % (n)	66 (209)	55 (46)	p=0.082
- brain metastases, % (n)	28 (89)	16 (13)	p=0.022
- liver metastases, % (n)	16 (52)	13 (11)	p=0.490
- bone metastases, % (n)	46 (145)	33 (27)	p=0.032
- adrenal metastases, % (n)	13 (41)	5 (4)	p=0.038
- metastases in other locations, % (n) [2]	8 (27)	10 (8)	p=0.741

Abbreviations: SE: standard error of the mean.

[1] statistical comparison with the chi-square test for categorical, and t-test for numerical variables; significant results are highlighted.

[2] intrathoracic: lung and pleural; other locations: e.g. abdominal or cervical lymph nodes, kidneys, spleen, skin, pancreas.

Supplementary Table 2: Results of basic laboratory tests at diagnosis in the study population

All stage IV $EGFR^+$ NSCLC patients (n=401, 100%)	*de novo* St. IV (n=318)	secondary St. IV (n=83)	p-value [1]
blood NLR, mean (SE) [2]	5.98 (0.33)	5.54 (0.52)	p=0.750
blood haemoglobin in g/dl, mean (SE)	13.1 (0.1)	13.2 (0.2)	p=0.669
blood platelets /nl, mean (SE)	313 (7)	302 (13)	p=0.480
serum LDH in U/l, mean (SE)	295 (11)	228 (8)	p=0.006
serum CRP in mg/dl, mean (SE)	22 (2.1)	15 (2.9)	p=0.125
serum creatinine in mg/dl, mean (SE)	0.83 (0.02)	0.84 (0.02)	p=0.978
serum GPT in U/l, mean (SE)	31 (1.6)	27 (2.0)	p=0.198

Abbreviations:SE: standard error of the mean; NLR: neutrophil-to-lymphocyte ratio.

[1] statistical comparison were performed with a t-test, and statistically significant results are highlighted.

[2] values for the various laboratory parameters available for 239-258/318 and 59-66/83 patients in the two groups.

Supplementary Table 3: The subset of patients after surgery and adjuvant chemotherapy

	de novo stage IV (n=318, see Table 1)	secondary stage IV, surgery & adj. CHT (n=33, Table 2)	p-value [1]
$EGFR^{mut}$ (del19/L858R/other), % (n)	60 (192) / 28 (88) / 12 (38)	52 (17) / 33 (11) / 15 (5)	p=0.611
TP53 co-mutation, % (n)	43 (90/211)	46 (10/22)	p=0.801
any co-mutation, % (n)	59 (124/211)	73 (16/22)	p=0.203
TKI PFS, median in months (IQR)	12 (7-21)	17 (10-26)	p=0.372
OS, median in months (IQR)	25 (20-29)	34 (19-48)	p=0.590

Abbreviations: adj. CHT: adjuvant chemotherapy; del19: exon 19 deletions; OS: overall survival; IQR: interquartile range; TKI: tyrosine kinase inhibitor.

[1] statistical comparison with the chi-square test for categorical, and with the logrank test for survival data.
